# Supplementary material for: Serum CD133-Associated Proteins Identified by Machine Learning Are Connected to Neural Development, Cancer Pathways, and 12-Month Survival in Glioblastoma
Source: Cancers (Basel). 2024 Aug 1;16(15):2740. doi: 10.3390/cancers16152740 (PMC11311306; doi:10.3390/cancers16152740)
Supplement: Supplementary file 1 [file cancers-16-02740-s001.zip › cancers-3087153-supplementary.pdf]

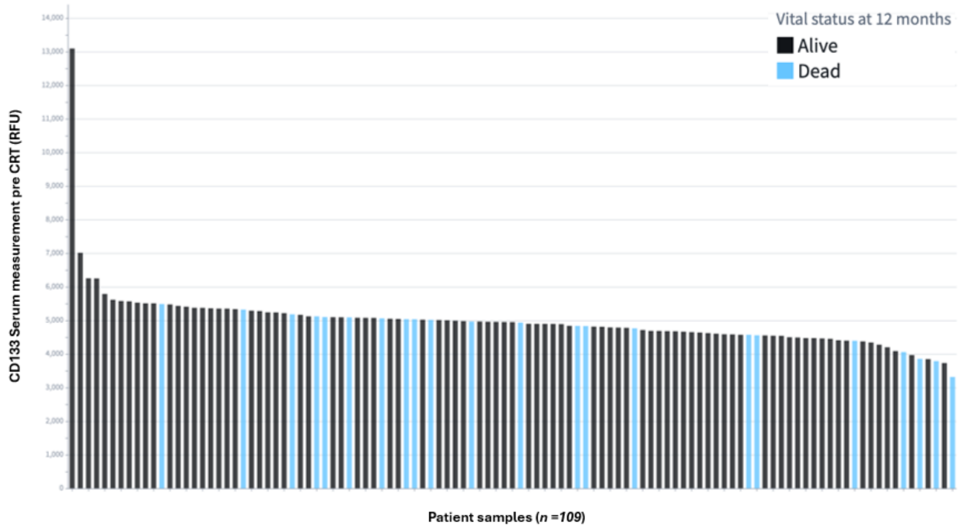

Supplemental Figure S1. Pre CRT serum CD133 measurements in RFU in relation to patient vital status at 12 months (black bars indicate that the patient is alive, blue bars indicate that the patient passed away prior to 12 months from diagnosis).

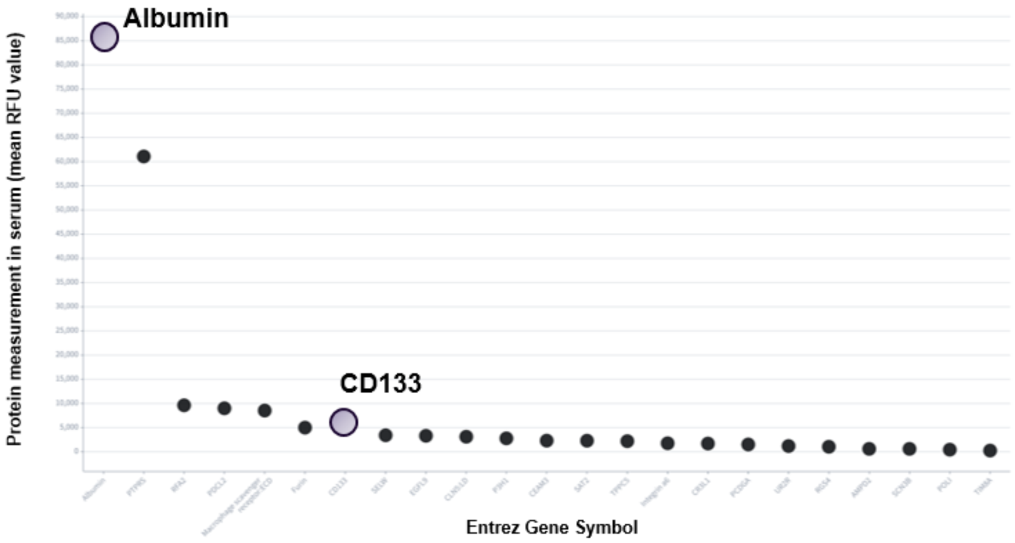

Supplemental Figure S2. Serum measurement levels of identified proteins in relationship to serum Albumin and serum CD133.

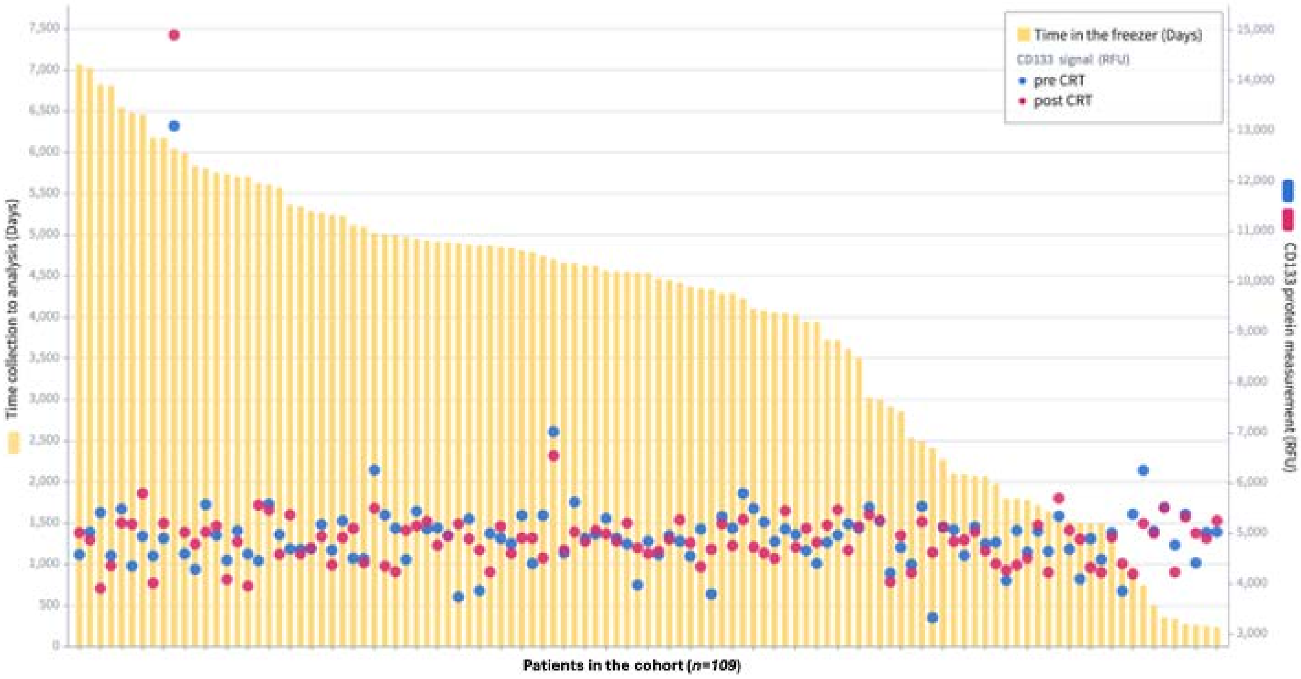

Figure S3. CD133 protein value based on the 7k proteomic panel in relation to the time the samples were stored in the freezer from collection to analysis in days showing the signal measured in the CD133 protein value pre- (blue) vs. post-CRT (magenta), and the number of days in the freezer for each patient (orange). The CD133 expression does not correlate with the number of days the sam-ples were stored in the freezer.

Supplemental Table S1. Complete Cox Proportional Hazards Model Results with parameter estimates, *p*-values, and hazard ratios.

Entrez Gene Symbol	Target Full Name	Parameter Estimate	p-value	Hazard Ratio (HR) (95% CI for HR)
RPA2	Replication protein A 32 kDa subunit	-6.67	0.04	0 (0 - 0.76)
AMPD2	AMP deaminase 2	-2.42	0.05	0.09 (0.01 - 1.00)
DLK2	Protein delta homolog 2	-2.26	0.09	0.1 (0.01 - 1.39)
NEGR1	Neuronal growth regulator 1	-2.43	0.1	0.09 (0 - 1.6)
PDCL2	Phosducin-like protein 2	2.92	0.16	18.63 (0.33 - 1053.82)
POLI	DNA polymerase iota	-4.14	0.16	0.02 (0 - 4.94)
CEACAM3	Carcinoembryonic antigen-related cell adhesion molecule 3	2.6	0.23	13.42 (0.19 - 957.60)
ITGA6	Integrin alpha-6	-5.27	0.31	0.01 (0 - 147.73)
PCDHGA10	Protocadherin gamma-A10	1.44	0.34	4.21 (0.22 - 81.21)
SELENOW	Selenoprotein W	1.38	0.36	3.99 (0.21 - 76.45)
TIMM8A	Mitochondrial import inner membrane translocase subunit Tim8 A	0.82	0.46	2.28 (0.26 - 19.81)
CLN5	Ceroid-lipofuscinosis neuronal protein 5:Lumenal domain	-2.95	0.53	0.05 (0 - 546.31)
IL15RA	Interleukin-15 receptor subunit alpha	-0.55	0.59	0.58 (0.08 - 4.32)
P3H1	Prolyl 3-hydroxylase 1	-2.87	0.61	0.06 (0 - 3045.74)
UTS2R	Urotensin-2 receptor	-1.18	0.61	0.31 (0 - 29.41)
RGS4	Regulator of G-protein signaling 4	-1.69	0.69	0.18 (0 - 864.82)
PTPRS	Receptor-type tyrosine-protein phosphatase S	0.71	0.71	2.04 (0.05 - 87.94)
FURIN	Furin	-0.61	0.72	0.54 (0.02 - 16.40)
MSR1	Macrophage scavenger receptor types I and II:Extracellular domain	-0.24	0.75	0.79 (0.19 - 3.32)
CSNK2B	Casein kinase II subunit beta	0.33	0.8	1.39 (0.1 - 19.43)
SAT2	Diamine acetyltransferase 2	-0.61	0.84	0.54 (0 - 201.37)
TRAPPC5	Trafficking protein particle complex subunit 5	0.6	0.86	1.82 (0 - 1319.35)
CREB3L1	Cyclic AMP-responsive element-binding protein 3-like protein 1	-0.33	0.88	0.72 (0.01 - 48.87)
SCN3B	Sodium channel subunit beta-3	-0.01	0.99	0.99 (0.13 - 7.79)
